# The DRUID study: racism and self-assessed health status in an indigenous population

**DOI:** 10.1186/1471-2458-12-131

**Published:** 2012-02-14

**Authors:** Yin C Paradies, Joan Cunningham

**Affiliations:** 1 McCaughey Centre, Melbourne School of Population Health, University of Melbourne, Melbourne, Australia; 2 Menzies School of Health Research, Charles Darwin University, Darwin, Australia and School of Population Health, University of Melbourne, Melbourne, Australia

## Abstract

**Background:**

There is now considerable evidence from around the world that racism is associated with both mental and physical ill-health. However, little is known about the mediating factors between racism and ill-health. This paper investigates relationships between racism and self-assessed mental and physical health among Indigenous Australians as well as potential mediators of these relationships.

**Methods:**

A total of 164 adults in the Darwin Region Urban Indigenous Diabetes (DRUID) study completed a validated instrument assessing interpersonal racism and a separate item on discrimination-related stress. Self-assessed health status was measured using the SF-12. Stress, optimism, lack of control, social connections, cultural identity and reactions/responses to interpersonal racism were considered as mediators and moderators of the relationship between racism/discrimination and self-assessed health status.

**Results:**

After adjusting for socio-demographic factors, interpersonal racism was significantly associated with the SF-12 mental (but not the physical) health component. Stress, lack of control and feeling powerless as a reaction to racism emerged as significant mediators of the relationship between racism and general mental health. Similar findings emerged for discrimination-related stress.

**Conclusions:**

Racism/discrimination is significantly associated with poor general mental health among this indigenous population. The mediating factors between racism and mental health identified in this study suggest new approaches to ameliorating the detrimental effects of racism on health. In particular, the importance of reducing racism-related stress, enhancing general levels of mastery, and minimising negative social connections in order to ameliorate the negative consequences of racism.

## Background

Racism is an organised system that labels some ethnic/racial groups as inferior to others and differentially allocates desirable societal resources to the superior ethnic/racial groups (Bonilla-Silva 1997). Racism can be defined as avoidable and unfair phenomena that result in inequality of resources, opportunities or benefits among racial/ethnic groups. It can be expressed through stereotypes (racist beliefs), prejudice (racist emotions) or discrimination (racist behaviours and practices) [1].

Regardless of its health, social or economic effects, racism is first and foremost a breach of human rights as enshrined in various national and international laws. Nonetheless, racism is also a social determinant of health that we need to understand in order to inform programs and policies to ameliorate its detrimental effects [2].

In recent years, the study of racism and health has emerged as an area of public health research. At the end of 2007, reviews identified over 250 publications examining racism as a determinant of mental and physical health and/or health behaviours, revealing strong associations between self-reported racism and ill-health among minority groups in developed countries [3-5]. These associations remained after adjusting for a range of confounders and occurred in longitudinal as well as cross-sectional studies, suggesting that racism precedes ill-health rather than vice versa. The most consistent finding has been the association between racism and mental ill-health [3-5].

It is important to note that this body of literature has predominately focused on the relationship between self-reported (also known as perceived) racism and health. This constitutes only one avenue through which racism influences health. More generally, racism can act through a number of pathways to affect mental and physical health, including via: reduced access to health-promoting social determinants of health such as education and employment, induced acute and chronic stress, reduced self efficacy, self esteem, increased uptake of substance use, higher rates of self harm, reduced quality and quantity of actual and perceived social support, and detrimental effects on cultural identity [6,7].

Exposure to racism as a form of stress can give rise to a range of factors that contribute to ill-health [8,9]. These factors include negative emotional states, reduced self-esteem, low self-efficacy and reduced self-control as well as pessimism, aggression, hyper-vigilance, and rumination [3-5]. Racism has also been linked to hypertension [10], cortisol dysregulation [11-13], sleep disturbance [14,15], obesity [16-18], and smoking [19-23] as well as alcohol and illicit drug use [23-28]. These factors have, in turn, been associated with poor mental and physical health [5,29-31].

To date, racism and health literature has focused primarily on the United States and specifically African Americans [3,4]. In addition, little is known about the causal pathways leading from racism to ill-health [32]. This paper adds to existing scholarship by examining the relationship between racism and self-assessed health in an indigenous population outside the United States-in particular, Indigenous Australians.

Indigenous (also known as Aboriginal) Australians constitute 2.4 per cent of the Australian population and suffer high rates of unemployment and incarceration, low income, sub-standard housing and a high burden of ill-health and mortality, including a life expectancy 9-12 years less than other Australians [33]. The poor health of Indigenous Australians has been associated with experiences of racism in several previous studies [34,35].

This disadvantage is associated with both historical and contemporary racism, colonisation and oppression [34]. Data from a 2008 national survey indicate that 27% of Aboriginal and Torres Strait Islanders aged 15 years and over report experiencing racism in the past 12 months [36]. Previous studies exploring the health effects of racism for Indigenous Australians identify associations between self-reported racism and poor health, including: self assessed health status [37,38], physical [39,40] and mental health outcomes [34,35,39,41-43] as well as substance use [34,44].

This paper focuses on interpersonal rather than institutional and/or systemic racism. It is acknowledged, however, that systemic racism is likely to have a stronger and more pervasive influence on health both directly and indirectly through upstream determinants (e.g. socioeconomic status). As well as focusing on interpersonal racism and health in an under-studied population, this paper adds to the scant literature examining factors through which racism may influence health. Research on such mediating factors and the causal pathways through which they operate can help identify "new options for coping with racism" [9]. We examined several variables as potential mediators of the relationship between racism and health, including: stress, optimism, social connections, lack of control, cultural identity and reactions/responses to interpersonal racism.

## Methods

Data for this study was drawn from the Darwin Region Urban Indigenous Diabetes (DRUID) Study. Those eligible were adults aged 15 years or more who self-identified as Indigenous and who lived in a private dwelling in a defined geographical area in and around the city of Darwin, Australia [45]. DRUID was a cross-sectional study in which participants were recruited through community and family networks as well as local health centres/departments [45]. As such, the study sample is not necessarily representative of the eligible population. The study utilised questionnaires to assess health status, socio-demographics, and psychosocial and behavioural factors. Of the 164 respondents included in this analysis, 77% (126) completed the DRUID survey with the assistance of an interviewer while 23% (38) self-administered the survey. The study was approved by the joint Human Research Ethics Committee of Menzies School of Health Research and Northern Territory Department of Health and Community Services.

### Measures

The Measure of Indigenous Racism Experiences (MIRE) was used to assess racism [46]. Respondents were asked how often they were treated unfairly because they are Indigenous across nine mutually exclusive items covering experiences in employment, domestic, educational/academic, recreational/leisure, law (enforcement), health care, government and non-government service provision (e.g. restaurants, bars, shops, banks) as well as general public settings (e.g. on the street, at shopping centres, sporting events, concerts, nightclubs etc.) [46]. Possible responses were: never (coded as 0), hardly ever (coded as 1), sometimes (coded as 2), often (coded as 3), very often (coded as 4) and 'not applicable'. The MIRE data examined in this paper appeared towards the end of the DRUID survey. Questions on health history, health behaviours, socioeconomic status, culture and identity, self-assessed health status and stressful experiences were asked prior to the MIRE questions, while questions on depression, lack of control, social support and optimism/pessimism were asked after the MIRE questions.

As part of a larger stress checklist (described below), respondents were also asked if they or their family/friends had experienced discrimination (not specifically attributed to race/ethnicity) that had affected them personally in the past 12 months ('discrimination-related stress'). Participants could respond that the event: was not experienced, had minimal/no effect, affected them somewhat, or affected them a great deal.

Age, sex, marital status and household composition (only Indigenous members or both Indigenous and non-Indigenous members) were assessed along with level of education, gross weekly household equivalised income using the modified OECD scale [47] and housing tenure. Five reactions and six responses to racism were also assessed as mediators. Reactions included: feeling ashamed, humiliated, anxious or fearful (labeled as 'ashamed'); feeling angry, annoyed or frustrated ('angry'); feeling amused, contemptuous or sorry for the person who did it ('amused'); feeling powerless, hopeless or depressed ('powerless'); and getting a headache, an upset stomach, tensing of your muscles, or a pounding heart ('somatic'). Responses included: ignoring, forgetting or accepting racism ('ignoring'); avoiding racism ('avoiding'); changing self or actions to prevent racism ('changing'); doing something about perpetrators or racist situations ('acting'); talking to family/friends or expressing racist experiences ('talking'); and keeping it to oneself ('withdrawing'). These items could be answered as: never, hardly ever, sometimes, often, or very often [46].

Stress over the past 12 months was assessed using a scale constructed *de novo *for the DRUID study. This included 10 items about acute stress (one-off, sudden or unexpected events including relationship breakup, death of partner/child, being fired, witness/target of violence, family member sent to jail, serious illness or accident/injury as well as other unspecified events) and 20 items about chronic stress (regular or continuing events including trouble at work, school or relationships; problems with alcohol/drugs, gambling, money, mental/emotional, health, employment, police or access to land/country; abuse/violence, overcrowding; family in prison; pressure to fulfill cultural or family/carer responsibilities; worry about family; reliance on others for everyday needs as well as other unspecified events). Each of these items assessed stress experienced either personally or in relation to the respondent's family and close friends (i.e. vicarious stress). These 30 items were assessed using a four-point response scale (event not experienced, had minimal/no effect, affected me somewhat, or affected me a great deal).

Optimism was assessed using the Life Orientation Test-Revised (LOT-R) [48]. This scale includes six items assessing optimism in uncertain times, expectations that things will go wrong for the respondent, optimism about their future, whether they generally expect things to go their way, the degree that they count on good things happening to them and whether they expect more good than bad things to happen to them. Lack of control was assessed via four items (how much, in the past four weeks you felt a lack of control with: life in general; finances; personal life; and health) with responses: never/rarely, sometimes, often, or very often (d'Espaignet, Measey, & Dal Grande, 2002).

Positive, negative and instrumental social connections were assessed using scales adapted from the Strong Heart Study [49]. Positive social connections included six items assessing the extent to which friends or relatives cared about, understood, or appreciated the respondent as well as to what degree he/she could rely upon, talk to, and relax around friends/relatives (not much, some and a lot). Negative social connections included five items assessing the degree to which friends/relatives made too many demands, argued with, criticised, let down or got on the nerves of the respondent (rarely/never, sometimes and often). Instrumental social connections were assessed with five items asking if there was someone the respondent could: go with to community/social events; borrow money from in an emergency; lend them a car or drive them somewhere if needed; ask to provide bail; and count on to check in on the respondent regularly (no; yes, one person or a few people; or yes, lots of people). As measures of cultural identity, participants were asked if they recognise a homeland/traditional country and whether they identify with a clan, tribe or language group.

Self-assessed health status was assessed using the Medical Outcomes Study Short Form-12 (SF-12) version 2 [50]. This self-report scale assesses health status via mental and physical component scores (MCS and PCS, respectively) and has established psychometric, clinical and predictive validity across a range of populations [51-54], including urban Indigenous Australians [55]. The SF-12 was scored using established methods [50].

### Analytical approach

All analyses were conducted using Stata 10.1 for Windows (Stata Corporation, College Station, Texas: 2007). Of the 363 DRUID participants recruited during the period in which the MIRE was administered (Sept 2003-Mar 2004), 312 completed at least some of the MIRE. Missing data across socio-demographic and psychosocial variables (see below) reduced this to a sample of 164 participants with complete data. The majority of those excluded were missing information on income (n = 65) and interpersonal racism (n = 52), with 20-30 also missing data for each of the psychosocial variables. Excluded participants were similar to those included across socio-demographic variables, with the exception that included participants had a higher level of education. Participants who were older, female and of higher socioeconomic status were over-represented in this study compared to the Darwin or total Australian Indigenous populations [45]. As recommended by Sterne et al. [56], we did not utilise multiple imputation methods because data were not missing at random (analyses not shown).

As the internal reliability of the nine racism items was good (α = 0.83), responses were given values from zero (never) to four (very often) and summed, giving a potential range of 0-36. As the internal reliabilities of the acute stress items (α = 0.66), chronic stress items (α = 0.82) and total stress items combined (α = 0.85) were adequate to good, weighted summary scores were also calculated for each of these constructs. The four responses were given numerical values from zero (no experience) to three (affected me a great deal) and summed across items giving a potential range of 0-30, 0-60 and 0-90 for acute, chronic and total stress, respectively. Positive (α = 0.84), negative (α = 0.75) and instrumental (α = 0.77) social connections were also summed and combined into composite variables. Both optimism (α = 0.58) and lack of control (α = 0.81) were coded as composite variables such that larger values represented higher levels of optimism and increased lack of control, respectively. The cultural identity items were coded dichotomously.

SF-12 MCS and PCS were examined as dependent variables in separate hierarchical regression models with racism as the independent variable. Even under various transformations, both the SF-12 MCS and PCS were significantly non-normal. As a result, these variables were dichotomised at the median (0 = lower quantile, 1 = upper quantile) and were also coded separately as tertiles. As a form of sensitivity analysis, binary MCS and PCS variables were examined with logistic regression models while trinary-coded MCS and PCS variables were examined using both multinomial and ordinal models.

Age, sex and racism were included in the first step of the regression models, with additional socio-demographic variables (household composition, marital status, equivalised household income, housing tenure and level of education) added in the second step. Psychosocial variables (acute, chronic and total stress, optimism, lack of control, the three types of measures of social connections and the two cultural identity variables) were entered in the third step. The likelihood ratio test was used to assess significance for each step of the analysis. Variance inflation factors of less than two across all models indicated that multicollinearity was not present. Socio-demographic or psychosocial variables with significant main effects were examined as potential moderators in a model that included mental/physical health status, racism, the variable in question and its interaction with racism.

These same psychosocial variables plus responses/reactions to racism were assessed separately as potential mediators of the association between racism (as the independent variable) and mental/physical health status (as the dependent variable). Given that mediated effects are unlikely to be normally distributed, bias-corrected non-parametric percentile confidence intervals were utilised. Because the forced symmetry of ordinary confidence intervals can result in estimation inaccuracies, bias-correct confidence intervals are adjusted to the percentile values of the sorted distribution of bootstrap estimates used for determining the bounds of the interval [57].

Bootstrapping with 5,000 replications was undertaken in order to provide increased statistical power [57]. As variables to be examined as potential mediators were non-normal (even under various transformations), they were dichotomised at the median and analysed using binary mediation with probit models [58,59]. Mediators found to be significant in models including only a single mediating variable between racism and mental/physical health status (adjusted for age, sex, household composition, marital status, equivalised household income, housing tenure and level of education) were then entered simultaneously in a multiple mediation model that adjusted for this same set of potential confounders. Separate models were run with both mental (SF-12 MCS = 1) and physical health (SF-12 PCS = 1) as the dependent variables.

In the results, the proportion of an association between the independent and the dependent variable accounted for by a specific mediator is expressed as a percentage. This proportion is calculated as variance in the dependent variable explained by the mediator (i.e. indirect effect) divided by sum of: (1) the variance in the dependent variable explained by the independent variable (i.e. direct effect); and (2) the variance in the dependent variable explained by the mediator (i.e. indirect effect/(indirect effect + direct effect)).

All models were also run using discrimination-related stress in place of interpersonal racism as the independent variable. Although this item assessed discrimination generally (including vicariously) rather than discrimination specifically attributed to race/ethnicity, duplicating analysis using this variable constitutes a form of sensitivity analysis that serves to assess the robustness of the findings relating to interpersonal racism (as the primary independent variable of interest). Consonant findings across these two variables indicate that spurious findings resulting from the multiple comparisons undertaken are not a concern (i.e. similar findings mitigate against Type I error).

## Results

Table 1 summarises participant characteristics. Participants ranged in age from 16-81 years (mean 39.7) and were predominantly female. The mean interpersonal racism score of 5.5 equates to approximately a quarter of respondents reporting no racism, half reporting 'hardly ever' experiencing racism and the remaining quarter reporting racism 'sometimes', 'often' or 'very often' on average across nine items. All participants included in the analysis reported experiencing racism in at least one setting. About a quarter of respondents reported racism in only one setting, a third in two settings, a quarter in three settings and a fifth in four or more settings. Respondents reported a range of reactions/responses to racism, such that no particular response was rare. Reported by over 80% of respondents, the most common reactions were feeling angry and amused and the most common responses were ignoring or talking about racism. Further details on the prevalence of reactions/responses to racism are detailed elsewhere [41].

**Table 1:** Characteristics of DRUID participants (n = 164 for categorical variables)

Categorical variables	Number*	%*
Sex		
Male	53	32
Female	111	68
Age group (years)		
16-24	26	16
25-34	36	22
35-44	42	26
45-54	38	23
55-64	15	9
65+	7	4
Marital status		
Never Married	38	23
Married/de facto	88	54
Separated/divorced/widowed	38	23
Gross weekly household equivalised income ($AU)		
1-199	42	26
200-499	63	38
500+	59	36
Household tenure		
Owned/being purchased	82	50
Rented/other	82	50
Household composition		
All Indigenous members	97	59
Indigenous and non-Indigenous members	67	41
Level of education		
Less than Year 10	34	21
Year 10/12	41	25
Non-degree qualifications	65	40
Degree	24	15
Identifies with a clan, tribe or language group		
Yes	118	72
No	46	28
Recognises homeland/traditional country		
Yes	129	78
No	35	22
Discrimination-related stress		
Yes	121	74
No	43	26
Composite variables	Range	Mean (SD)
Interpersonal racism	0-24	5.5 (5.4)
10-item acute stress	0-18	4.8 (4.4)
20-item chronic stress	0-52	11.9 (8.6)
30-item total stress	0-67	15.8 (11.9)
Optimism	12-30	21.0 (4.1)
Lack of control	0-12	3.4 (2.9)
Positive social connections	6-18	15.3 (2.9)
Negative social connections	5-13	8.2 (2.2)
Instrumental social connections	1-10	6.2 (2.0)
Scored variables	Range	Median (SD)
SF-12 Mental Component Score	19.7-65.8	47.9 (10.5)
SF-12 Physical Component Score	18.1-66.1	51.0 (9.1)
Individual MIRE items (n = 164)	Range	Mean (SD)
Employment	0-4	0.76 (1.08)
Domestic	0-4	0.44 (0.85)
Educational/academic	0-4	0.56 (0.97)
Recreational/leisure	0-4	0.49 (0.83)
Law (enforcement)	0-4	0.48 (0.89)
Health care	0-3	0.38 (0.75)
Government service provision	0-3	0.51 (0.89)
Other service provision	0-4	0.93 (1.05)
Public settings	0-4	0.93 (0.99)

* Note: Percentages may not add to 100 due to rounding. SD (Standard Deviation)

Neither interpersonal racism nor discrimination-related stress emerged as significant predictors SF-12 PCS in any regression models. Similarly, no significant findings were produced from mediation analysis with PCS as the dependent variable. Regression models with SF-12 MCS as the dependent variable and interpersonal racism as the independent variable are shown in Table 2. Interpersonal racism accounted for 8% of the variance in mental health (although included in model 1, analyses not shown indicate that age and sex accounted for none of this variance). Household composition, level of education, equivalised household income, housing tenure and marital status accounted for 7% of the variance in mental health. The psychosocial variables added in model 3 together explained another 29% of the variance. The likelihood ratio test was significant at each step of the analysis. Although negative social connections were significant in model 3 (Table 2, Odds Ratio: 0.82, CI 0.67-0.999), the interaction between racism and this variable was not significant.

**Table 2:** Hierarchical regression models with SF-12 MCS = 1 as the dependent variable#

	Model 1 OR (95% CI)	Model 2 OR (95% CI)	Model 3 OR (95% CI)
Interpersonal racism	**0.91 **(0.86-0.97)	**0.90 **(0.84-0.96)	0.98 (0.89-1.07)
Age	1.00 (0.98-1.03)	1.00 (0.96-1.03)	0.99 (0.96-1.03)
Female	0.63 (0.32-1.26)	0.57(0.27-1.18)	0.63(0.26-1.52)
Mixed household		0.80 (0.35-1.82)'	0.78 (0.29-2.10)
Education			
Less than Year 10		1.00	1.00
Year 10/12		0.93 (0.31-2.74)	0.81 (0.23-2.80)
Non-degree		0.66 (0.23-1.86)	1.00 (0.29-3.45)
Degree		1.09 (0.30-3.95)	1.47 (0.31-6.95)
Household equivalised income			
"$1"-199		1.00	1.00
$200-499		1.25 (0.52-3.04)	0.57 (0.20-1.67)
$500+		1.97 (0.72-5.39)	0.76 (0.22-2.67)
Own/purchasing home		1.48 (0.71-3.10)	1.83 (0.78-4.31)
Marital status			
Never married		1.00	1.00
Married/defacto		1.46 (0.53-4.01)	1.96 (0.57-6.77)
Separated etc.		0.74 (0.22-2.52)	0.85 (0.21-3.48)
Acute stress			0.91 (0.82-1.02)
Chronic stress			0.97 (0.91-1.04)
Optimism			1.05 (0.95-1.17)
Lack of control			0.85 (0.71-1.00)
Negative social connections			**0.82 **(0.67-1.00)
Positive social connections			1.13 (0.95-1.33)
Instrumental social connections			1.04 (0.83-1.29)
Identifies with clan/tribal/language			0.83 (0.31-2.26)
Recognises homeland country			0.56 (0.17-1.77)
Constant	3.27 (0.73-14.68)	3.40 (0.46-25.19)	3.77 (0.05-273.5)
McKelvey-Zavoina R-squared [60]	0.084	0.154	0.440

# SF-12 MCS is split at the median with the lower quantile (poorer mental health) coded as 0 and the upper quantile (better mental health) coded as 1. Other variables are continuous except where indicated

Largely comparable results were produced in hierarchical regression models with discrimination-related stress (instead of interpersonal racism) as the independent variable (analyses not shown). One difference, however, was that lack of control emerged as a significant variable in the model that included all socio-demographic and psychosocial variables, with a significant interaction effect (i.e. moderation) between discrimination-related stress and this variable. Stratified analysis indicated that discrimination-related stress was only associated with poor mental health (*p *= 0.06) among participants reporting a lack of control in life (i.e. those above the median score).

Multinomial and ordinal regression models also produced similar results with the exception that positive social connections emerged as an additional significant variable in the full multinomial and ordinal regression models with discrimination-related stress as the independent variable (analyses not shown).

Models including interpersonal racism along with a single mediating variable produced several significant findings (see Table 3). Feeling 'powerless' as a reaction to racism was a statistically significant mediator along with the three composite stress measures, lack of control and negative social connections. In each separate model, a single mediator accounted for 22% (negative social connections) to 71% (total stress) of the association between racism and mental health. Models with discrimination-related stress as the independent variable also identified feeling 'powerless' as a significant mediator along with somatic reactions to racism and the three composite stress measures (see Table 4).

**Table 3:** Factors significantly mediating the association between interpersonal racism and mental health (single mediation models)

Mediator	β coefficient	Bias-corrected lower CI	Bias-corrected upper CI	% of effect mediated
Total stress	-0.265	-0.416	-0.097	71.4
Chronic stress	-0.197	-0.343	-0.058	56.8
Lack of control	-0.139	-0.247	-0.045	40.1
Powerless	-0.136	-0.250	-0.043	39.2
Acute stress	-0.126	-0.231	-0.040	36.6
Negative social connections	-0.074	-0.171	-0.001	22.2

**Table 4:** Factors significantly mediating the association between discrimination-related stress and mental health (single mediation models)

Mediator	β coefficient	Bias-corrected lower CI	Bias-corrected upper CI	% of effect mediated
Total stress	-0.188	-0.301	-0.076	92.4
Powerless	-0.111	-0.214	-0.024	74.8
Chronic stress	-0.134	-0.238	-0.044	85.9
Acute stress	-0.140	-0.244	-0.045	85.8
Somatic reaction	-0.071	-0.170	-0.004	48.7

In the multiple mediation model with interpersonal racism as the independent variable, acute stress and lack of control remained as significant mediators after simultaneous adjustment for other mediators (see Table 5). All the mediating variables in this model (powerless, acute and chronic stress, lack of control and negative social connections) together accounted for 94% of the association between racism and mental health. Feeling 'powerless' as well as acute and chronic stress emerged as significant mediators in the multiple mediation model with discrimination-related stress as the independent variable (see Table 6). All the mediating variables in this model (powerless, somatic reaction, acute and chronic stress) together accounted for 75% of the association between discrimination-related stress and mental health.

**Table 5:** Factors mediating the association between interpersonal racism and mental health (multiple mediation model)

Mediator	β coefficient	Bias-corrected lower CI	Bias-corrected upper CI	% of effect mediated
Lack of control	-0.088	-0.200	-0.011	22.1
Acute stress	-0.086	-0.188	-0.012	21.5
Chronic stress	-0.095	-0.229	0.043	23.8
Powerless	-0.086	-0.194	0.121	21.5
Negative social connections	-0.021	-0.100	0.047	5.4
Total indirect effect	-0.377	-0.538	-0.182	94.3

**Table 6:** Factors mediating the association between discrimination-related stress and mental health (multiple mediation model)

Mediator	β coefficient*	Bias-corrected lower CI	Bias-corrected upper CI	% of effect mediated
Powerless	-0.074	-0.175	-0.007	19.9
Chronic stress	-0.090	-0.190	-0.020	24.3
Acute stress	-0.095	-0.194	-0.015	25.8
Somatic reaction	-0.017	-0.110	0.054	4.7
Total indirect effect	-0.276	-0.414	-0.129	74.7

## Discussion

This study found that after adjustment for socio-demographic factors, interpersonal racism is associated with mental but not physical health in a non-U.S. indigenous population. Stress, lack of control and feeling powerless as a reaction to racism emerged in multiple mediation models as significant mediators of the relationship between racism and general mental health.

To our knowledge, this is only the third study to show that racism is associated with general mental health among an indigenous population [35,38]. A study among 153 Indigenous Australians found that experiencing regular racism was associated with poor mental health (MCS SF-12) after adjusting for gender, age, education, employment and financial stress, but no association with physical health (PCS SF-12) was evident [35]. Some previous studies have similarly found that racism is only significantly associated with the mental but not physical health scale of the SF-12/36 [35,61-64] while others identified an association with the physical health scale [39,65-68]. Although the aetiology behind such differences is not yet understood, stronger associations between racism and mental (as opposed to physical) health have been noted worldwide [3-5]. As suggested in recent reviews, it is likely that racism impacts primarily on mental health, with physical health effects mediated through mental processes [8,9].

To our knowledge, no previous study has examined the factors included in this study as mediators/moderators of the relationship between racism and physical health. Lack of control is now well-established as a determinant of morbidity and mortality [69,70]. Only two previous studies have shown that lack of control acts as a mediator of the association between racism and mental health (see below) while control beliefs failed to emerge as a mediator between racism and depression in another study [71]. In a study involving 108 Arab-Americans, lack of control completely mediated the association between racism and self-esteem, and partially mediated the association between racism and psychological distress [72]. Similarly, mastery (i.e. the opposite of lack of control) mediated the relationship between racism and psychological distress among 485 African-American, Native-American and Asian-Americans [73]. Racism may lead to lack of control by creating unfair and unpredictable demands as well as attenuating rewards resulting from effort, with one study finding that increased reporting of racism among Latino students was associated with lack of control [74]. In one previous study of racism and health among Indigenous Australians, mastery failed to emerge as a significant mediator [40]. To our knowledge, this is the first study to suggest that lack of control may act as an effect modifier between discrimination and poor mental health, with discrimination only affecting only those low in control.

Eleven studies have examined social support, connections or capital as a mediator/moderator of the association between racism and mental health, with some evidence that it ameliorates the detrimental effect of racism on health [5,75]. This study, however, is the first to examine the role of negative social connections, finding it to be a significant mediator (but not moderator) of the relationship between racism and poor health when considered alone in a single variable mediator model.

It is possible that racism perpetrated by friends or relatives may take the form of demands, arguments, criticism, being let down or annoyance. Alternatively, or in addition, racism-related stress may precipitate negative social connections or reduce the capacity of individuals to tolerate social connections (hence increasing the reporting of negative connections). This latter possibility is supported by a study which found racism to be associated with increased reporting of routine social interactions as harassing, exclusionary, and unfair [76]. The failure of this mediator to emerge in the multiple mediation model suggests that it may act as a second-order mediator in a causal pathway with stress as the primary mediator.

Although two previous studies have found that improved mental health outcomes are associated with active rather than passive coping responses to racism [77,78], none of the six responses to racism mediated the association between racism and mental health in the DRUID study. However, powerlessness as a reaction to racism was found to be a significant mediator. This finding is particularly disturbing given that among a nationally representative group of Indigenous Australians, almost a third (28%) reported feeling powerlessness as a reaction to racism [34]. Somatic reaction was significant in single variable mediation models but was no longer so in the multiple mediation model. Although no previous research has examined reactions as mediators of the relationship between racism and ill-health, a study involving 183 Indigenous Australians found that racism which evoked an emotional/physical response was related to poor general health [39].

A previous study involving 215 multiethnic college students found that optimism was inversely associated with depression in a model that also included perceived racism [79]. However, this construct failed to emerge as a statistically significant mediator between racism and mental health in the DRUID study. Given that no other studies have specifically focused on optimism as a mediator, it is not yet clear how this construct may contribute to the aetiology of racism as a determinant of health.

Reviews in this field suggest that stress acts as a mediator between racism and poor health [3-5]. However, only a few studies have examined such a role, with stress acting as mediator between racism and: smoking [21], depression/anxiety [80,81], hypertension [82] and psychological well-being [75] while failing to emerge as a mediator between racism and depression [83]. Both acute and chronic stress emerged as important mediators in this study, remaining significant in multiple mediation models (with the exception of chronic stress in the interpersonal racism model). This supports the view that racism acts (at least in part) as a form of stress that, in turn, leads to both physical and mental ill-health through various psychological and physical consequences (e.g. allostatic load) [84].

Although four studies have found that ethnic identity buffers racism-related stress [75,80,85,86], a recent review of 12 studies notes that identity is not sufficient to completely ameliorate the effects of racism on health [87]. It is not clear why the two aspects of cultural identity assessed in this study failed to emerge as either significant mediators or moderators of the association between racism and mental health.

Results from models with interpersonal racism and discrimination-related stress were largely consistent, providing confidence that findings were not spurious. There were, however, some differences in findings across these two independent variables. In particular, discrimination-related stress was only associated with poor mental health among participants reporting a lack of control in life while no moderation was detected for interpersonal racism. While four significant mediators emerged in common across these two variables in single-mediation models, lack of control and negative social connections mediated only in models with interpersonal racism while somatic reactions mediated only in the model with discrimination-related stress as the independent variable. In multiple mediation models, acute stress was significant in both models. Lack of control was only significant in interpersonal racism model while powerlessness and chronic stress were significant in the discrimination-related stress model.

Variations in findings across the two measures of exposure may be related to the fact that discrimination-related stress assessed experiences not specifically attributed to race/ethnicity. Previous studies have found that association with health can vary across different measures of discrimination [88-90]. Further work is required to understand the nature and implications of these differential associations. Differences may also be due to the fact that discrimination-related stress also included assessment of vicarious as well as personal experiences of racism (i.e. racism experienced by family/friends). Although research on vicarious racism is very limited [3-5], there is some evidence of its detrimental effects on health and wellbeing [40].

The robustness of study findings is supported by broadly similar findings for both interpersonal racism and discrimination-related stress. Nonetheless, there are several study limitations to note. First, in terms of measurement, it is evident that racism can go unnoticed when it does occur and be perceived when it is not 'objectively' present (i.e. an avoidable and unfair inequality of resources, opportunity or benefit has not, in fact occurred). However, as with stress, perceived racism may act as a determinant of health regardless of its objective veracity [3-5].

The MIRE captures only those experiences of racism that respondents perceive and are willing to report. There is evidence that respondents are more likely to underestimate than overestimate experiences of racism [91-94]. This is due to a combination of factors, including the poorly understood (and largely invisible) nature of systemic racism, the protective effects that may accrue from not attributing experiences to racism [95], and the negative social repercussions of labelling an experience as racism [94].

Although 32 longitudinal studies suggest that the primary direction of causation is from racism to ill-health rather than ill-health leading to increased reporting of racism [3-5,96-98], the latter cannot be ruled out in this cross-sectional study.

Failure to identify any significant moderators may have been due to the low power of moderation tests [99] combined with a relatively small sample size. Similarly, low power due to both the small sample size and the need to dichotomise mediators may have precluded identification of weaker mediation effects [100].

With figures ranging from 0.60-0.80 in the literature [101,102], there is no established cut-off for satisfactory reliability as measured by Cronbach's alpha. Nonetheless, it is clear that two of the composite measures in this study had low to moderate internal consistencies (acute stress: α = 0.66 and optimism: α = 0.58). As such, these scales may have not have tapped into their respective underlying constructs with sufficient accuracy. While the optimism scale (LOT-R) has well established internal consistency [103], the acute stress scale is novel to DRUID and its psychometric properties should be examined in further research.

## Conclusions

This study highlights the complex pathways leading from racism to mental ill-health and elucidates several new avenues for further research and practice. It is notable that the mediating variables examined here together accounted for 75% and 94% of the association between discrimination-related stress and interpersonal racism and mental health, respectively. This indicates the importance of understanding the aetiology of racism as a determinant of health. In addition to reinforcing the clear need to reduce the incidence of racism [104], such an improved understanding may help direct efforts to combat the effects of racism. In particular, this study highlights the importance of reducing consequent stress, enhancing general levels of mastery, and minimising negative social connections in order to ameliorate the negative consequences of racism.

## Competing interests

The authors declare that they have no competing interests.

## Authors' contributions

YP was involved in designing the study, conducted the data analysis and wrote the manuscript. JC was involved in the design and coordination of the project, participated in data collection, advised on data analysis and edited the manuscript. All authors read and approved the final manuscript.

## Pre-publication history

The pre-publication history for this paper can be accessed here:

http://www.biomedcentral.com/1471-2458/12/131/prepub
